# TRPV4-mediated trigeminal pain: behavior assessments and mechanisms

**DOI:** 10.1186/1744-8069-10-S1-P1

**Published:** 2014-12-15

**Authors:** Yong Chen, Farshid Guilak, Robert W Gereau IV, Fan Wang, Wolfgang Liedtke

**Affiliations:** 1 Department of Neurology, Duke University, Durham, NC 27110, USA; 2 Department of Orthopedic Surgery, Duke University, Durham, NC 27110, USA; 3 Department of Neurobiology, Duke University, Durham, NC 27110, USA; 4 Department of Anesthesiology, Washington University, St. Louis, MO 63110, USA

## 

Trigeminal pain represents one of the worst pains that humans can suffer. One of the obstacles towards development of rationally targeted therapies is rooted in shortcomings of available animal models for trigeminal pain. Another roadblock is lack of clear understanding of molecular and cellular mechanisms that underlie this type of pain.

TRPV4 is a polymodally activated Ca^2+^-permeable nonselective cation channel that is activated by a variety of factors, including chemical, osmotic, mechanical, moderate heat and low pH stimuli. Previous studies detected that it is highly expressed in trigeminal ganglion (TG) sensory neurons with small diameter, indicating it might function in trigeminally mediated pain.

We first demonstrated that the TRPV4 channel is critical for TMJ-inflammation evoked pain behavior in mice, and that TG pro-nociceptive changes are Trpv4-dependent. As a quantitative metric, bite force was recorded as evidence of masticatory sensitization, in keeping with human translational studies. In Trpv4^-/-^ mice with TMJ-inflammation, attenuation of bite force was significantly reduced compared to WTs. TMJ-inflammation and mandibular skeletal changes were apparent after CFA injections, but remarkably independent of Trpv4 genotype. Intriguingly, as a result of TMJ-inflammation, WT mice exhibited significant up-regulation of TRPV4 and phospho-ERK in TMJ-innervating TG neurons, absent in Trpv4^-/-^ mice. Mice with genetically impaired MEK/ERK phosphorylation in neurons showed a similar resistance to reduction of bite-force as Trpv4^-/-^ mice. Thus, TRPV4 is necessary for masticatory sensitization in TMJ-inflammation, and likely functions up-stream of MEK/ERK phosphorylation in TG neurons in-vivo.

Next, we tested whether TRPV4 ion channels might be critical for irritant-evoked trigeminal pain behavior. Our results demonstrate TRPV4 to be critically involved in trigeminal nocifensive behavior evoked by whisker-pad injections of formalin. We have used Trpv4^-/-^ mice and TRPV4-specific antagonists in mice to support this conclusion. Furthermore, our results imply TRPV4 to activate MEK-ERK in TG neurons. Importantly, cellular studies suggest that TRPV4 can be activated directly by formalin to gate Ca^2+^ ions.

Last, we developed a novel behavioral assay of water licking for assessing trigeminal irritant pain. We found that formalin-induced irritation in the V2 territory decreased the water licking times and increased the latency of first water licking in WT, which were significantly attenuated in Trpv4^-/-^ mice.

Taken together, our results imply that TRPV4 represents a novel pro-nociceptive target in trigeminal pain including TMJ, and thus a potential target for novel pain alleviating strategies for TMJ and other trigeminal pain disorders.

## Disclosures

None of the authors have conflicts of interest with respect to this work.

